# Preparation, Characterization and Anti-Complementary Activity of Three Novel Polysaccharides from *Cordyceps militaris*

**DOI:** 10.3390/polym14214636

**Published:** 2022-10-31

**Authors:** Zhengyu Hu, Jiaming Wang, Long Jin, Tieqiang Zong, Yuanqi Duan, Jinfeng Sun, Wei Zhou, Gao Li

**Affiliations:** 1 Key Laboratory of Natural Medicines of the Changbai Mountain, Ministry of Education, Yanbian University, Yanji 133002, China; 2 School of Pharmacy, Qiqihar Medical University, Qiqihar 161006, China

**Keywords:** *Cordyceps militaris*, anti-complementary activity, polysaccharides, structure-activity relationship

## Abstract

This investigation focuses on the three novel polysaccharides from *Cordyceps militaris* and then discusses their characterization and anti-complementary activity. The three polysaccharides from *C. militaris* (CMP-1, CMP-2 and CMP-3) were prepared using a DEAE-52 cellulose column. The HPLC, HPGPC, FT-IR and Congo red analyses were used to characterize their monosaccharides, molecular weight and stereo conformation, which demonstrated that the three polysaccharides were homogenous polysaccharides with different molecular weights and were composed of at least ten monosaccharides with different molar ratios, and all had a triple-helix conformation. The evaluation of anti-complementary activity demonstrated that the three polysaccharides significantly inhibited complement activation through the classical pathway and alternative pathway. Preliminary mechanism studies indicated that CMP-1, CMP-2 and CMP-3 acted with C2, C5, C9, factor B, factor B, and P components in the overactivation cascade of the complement system. The analysis of the Pearson correlation and network confirmed that the ribose, glucuronic acid and galacturonic acid composition were negatively correlated with the anti-complementary activity of polysaccharides. These results suggested that the three novel polysaccharides are potential candidates for anti-complementary drugs.

## 1. Introduction

The complement system is considered to be the chief component of innate immunity and plays an important role in host protection, consisting of more than 40 serine proteases, receptors and inhibitors. Complement has a strict mechanism of operation to avoid damage to autologous tissues and to clear pathogens under normal conditions. However, when complement is overactivated it can cause severe complement-mediated diseases [1,2,3]. Currently, drugs with known anti-complement abilities such as glucocorticoids and rituximab have been used in the treatment of diseases associated with hypercomplementation, but their clinical application is plagued by significant adverse effects and high prices [4]. Recently, natural polysaccharides, which are complement inhibitors with high efficiency and low toxicity, have garnered the attention of researchers, serving as potential agents of complement inhibitors [5,6,7]. These reported polysaccharides with complement inhibition are mainly from plants, while a few studies have been reported on the complement activity of polysaccharides from fungi.

*Cordyceps militaris* (L. ex Fr.) Link. is an entomogenous fungi that belongs to the genus *Codyceps* and the family Hypocreaceae, which is widely distributed in China, Japan, Korea and other East Asian countries [8]. It has been used as a healthy food and folk medicine for the treatment of malaria, palpitations, fever, dizziness and chronic kidney disease [9]. Various bioactive constituents from the *C. militaris* have been reported, including polysaccharides, cordycepin, volatile oils and flavonoids [10,11,12]. Polysaccharides are one of the most abundant and important components in *C. militaris*, which can exhibit a variety of various pharmacological effects, such as antioxidant, antitumor, anti-inflammatory, immunomodulatory, antihyperlipidemic, and hepatoprotective [13,14,15,16,17]. Especially, *C. militaris* polysaccharides (CMPs) can upregulate the expression of TNF-*α*, IFN-*γ*, and IL-1*β* mRNA in mice [18]. Simultaneously, the over-activation of the complement system is involved in many autoimmune disorders and inflammatory diseases [19], and is closely related to the above immune cytokines, which imply that the polysaccharide fractions with anti-complement activity may be present in *C. militaris*. However, no relevant studies on the preparation and characterization of polysaccharides with anti-complement effect from *C. militaris* have been reported.

The present study aimed to prepare novel polysaccharides with anti-complementary activity from *C. militaris*. Herein, three novel polysaccharides from *C. militaris* (CMP-1, CMP-2 and CMP-3) were successfully prepared by a DEAE-52 cellulose column. The structural characterization of the three polysaccharides were analyzed via chromatographic and spectroscopic techniques. The complement inhibition ability and their obstructed targets were evaluated by immune haemolysis test, and the further structure–activity relationship was preliminarily explored through the Pearson correlation and network analysis.

## 2. Materials and Methods

### 2.1. Fungus Material and Reagents

Cultured *Cordyceps militaris* was obtained from the Yanbian Foresty Science Institute (Yanji, China), in September 2020 and identified by Prof. Gao Li (College of Pharmacy, Yanbian University). The voucher specimen (voucher number: YB-YC-2032) has been deposited at the Pharmacognosy Laboratory of the College of Pharmacy, Yanbian University. The mannose (Man), glucosamine (GlcN), ribose (Rib), rhamnose (Rha), glucuronic acid (GlcA), galacturonic acid (GalA), glucose (Glc), galactose (Gal), xylose (Xyl), arabinose (Ara) and fucose (Fuc) were purchased from Shanghai Aladdin Biochemical Technology Co., Ltd. (Shanghai, China). The different complement components (C2, C3, C4, C5, C9, Factor B, Factor D, Factor P) were obtained from MineBio Life Sciences Ltd. (Shanghai, China). All reagents and solvents were of analytical grade. The Yanbian University Institutional Research Ethics Committee accepted all the experimental procedures and protocols (approval NO. YBU-2020-091801).

### 2.2. Preparation of Polysaccharides

The crude *C. militaris* polysaccharides (CMPs) were prepared by water extraction and the alcohol precipitation method. Briefly, the *C. militaris* was crushed into powder and pretreated with 80% ethanol (Shanghai Macklin Biochemical Technology Co., Ltd., Shanghai, China)three times, as described in our previous work [20]. The *C. militaris* residues were refluxed twice with deionized water (40:1, mL/g) at 80 °C for 60 min. The extraction solution was concentrated to 1/5 of the original volume, followed by precipitation through the addition 95% ethanol and refrigerated overnight to obtain polysaccharide precipitation. Furthermore, the precipitation of polysaccharides was redissolved with deionized water, and injected into a DEAE-52 cellulose column (26 mm × 300 mm), eluted with 0, 0.1, 0.2, 0.3, 0.4 and 0.5 mol/L NaCl, and determined at 490 nm by the phenol-sulphuric acid method to draw the elution curve [21]. The main eluent fraction was collected and dialyzed, and finally lyophilized for the next analysis.

### 2.3. Determination of Water Solubility Index

The water soluble index (WSI) of polysaccharides was determined by Ye et al. [22]. The dried sample (0.2 g) was mixed with deionized water (2 mL) for dissolution. Then, the mixture was centrifuged at 5000 rpm for 40 min. The precipitant was collected and lyophilized to a constant weight. The WSI was calculated as follows:

(1) $$\mathrm{WSI}\;\left(\%\right)=\left(M_{0}-M_{1}\right)/M_{0}\times 100\%$$

where M_0_ was the weight of the sample, M_1_ was the weight of the lyophilized precipitant.

### 2.4. Determination of Homogeneity and Molecular Weights

The homogeneity and molecular weights (Mw) of the sample were measured on a Waters high-performance gel permeation chromatography (HPGPC) equipped with a RI2000 refractive index detector (RID) (Schambeck SFD GmbH, Germany), and a Shodex sugar KS-804 column (8 mm × 300 mm, Showa Denko, Japan) [23]. The mobile phase was ultrapure water at a flow rate of 1.0 mL/min at 50 °C. The N2000 GPC system (Hangzhou Sno Scientifific Instrument Co. Ltd., Hangzhou, China) was used to process data. The molecular weights of the sample were calculated by the calibration curve obtained by using a series of dextran standards.

### 2.5. Determination of Monosaccharide Compositions

The monosaccharide composition of polysaccharides was analyzed by a high-performance liquid phase (Ultimate 3000 HPLC system, Thermo, Waltham, MA, USA) equipped with a diode array detector (DAD, Thermo, USA) [24]. The sample hydrolysate solution was hydrolyzed by trifluoroacetic acid (2 mol/L), added to 1-phenyl-3-methyl-5-pyrazolone solution (PMP, 0.5 mol/L) and NaOH solution (0.3 mol/L), and incubated at 70 °C for 60 min. The mixture was added to HCl solution (0.3 mol/L), and followed by extraction with chloroform for three times. The 20 µL of aqueous layer containing a PMP-labeled derivative was filtered, and injected into a Supersil ODS2 column (5 μm, 4.6 mm × 250 mm), and eluted at 0.8 mL/min with the mobile phase (acetonitrile and phosphate buffer solution, 18:82 *v*/*v*) at 30 °C. The detection wavelength was set as 245 nm. A series of diluted concentrations of standard monosaccharides were derived and analyzed for drawing standard curves to calculate the monosaccharide molar ratio of polysaccharides.

### 2.6. Determination of FT-IR

The FT-IR measurement (Gangdong Sci. & Tech. development Co. Ltd., Tianjin, China) was performed in the wavenumber range of 4000~400 cm^−1^ with KBr pellet (Dried polysaccharides: KBr powder, 2:100 mg/mg). Derivation including Savitsky-Golay algorithm with 13 smoothing factors was performed using the OMNIC 8.0 software incorporated into the instrument [25].

### 2.7. Congo Red Test

The triple helix structures of the samples were investigated using the Congo red, as described previously [26]. Briefly, the 2 mL of Congo red solution (80 μmol/L) and 2 mL of polysaccharides solution (2 mg/mL) were mixed with 1 mol/L the NaOH until the concentrations of NaOH in the mixtures were 0–0.5 mol/L. The absorption spectrum was scanned from 200~600 nm to obtain the maximum absorption wavelength (λ_max_)

### 2.8. Anti-Complementary Activity

#### 2.8.1. Anti-Complementary Activity through the Classical and Alternative Pathway

The anti-complementary activity of polysaccharides on the classical pathway (CP) was evaluated as described in our previous studies [27,28]. Briefly, the 6% sheep red blood cells were added with GVB-Ca^2+^/Mg^2+^ buffer solution to a constant volume until the concentration was 2 × 10^9^ cells/mL, then mixed with hemolysin in 1:1 ratio, incubated at 37 ℃ for 30 min, centrifuged to remove unconjugated hemolysin, and obtained sensitized sheep erythrocytes (EAs) for standby. The source of complement for the classical pathway was the normal healthy adult serum pool (NHSP). The polysaccharides solutions were prepared into different concentration gradients with GVB-Ca^2+^/Mg^2+^ buffer solution, then NHSP diluted in 1:80 was added, and EAs were added after 30 min incubation in a 37 ℃ water bath. The supernatant was collected through centrifugation, and absorbance was measured at 540 nm. The positive control was heparin. Finally, the polysaccharides concentration required for 50% hemolysis inhibition (CH_50_) was calculated according to the hemolysis inhibition rate of the different concentration gradients.

The anti-complementary activity of polysaccharides on the alternative pathway (AP) was determined to be similar to the above steps. The NHSP in GVB-Mg^2+^/EGTA as a complement source of the alternative pathway. In brief, the 2% rabbit erythrocytes (ERs) were mixed with GVB-Mg^2+^/EGTA buffer to a concentration of 5 × 10^8^ cells/mL. Then, NHSP and the diluted polysaccharide solution of different concentrations were pre-incubated for 15 min, and then ERs was added to incubate at 37 °C for 30 min. The reaction vessel was placed on ice for cooling until the reaction was terminated. The supernatant was obtained by centrifugation and placed in a 96 well plate, the absorbance value was scanned at 412 nm., and the 50% hemolytic inhibition concentration (AP_50_) was calculated in the alternative pathway.

#### 2.8.2. Identification of Complement Targets

The targets of polysaccharide in the complement cascade were identified by the previously reported method [29]. The critical concentration of polysaccharide was used as the test concentration of the complement target, and was selected to inhibit the concentration of polysaccharide when hemolysis was close to 100%. The different complement-depleted sera were incubated with EAs or ERs at 37 °C for 30 min. In the following operation steps, C2, C3, C4, C5 and C9 targets were identified as the same as the CP complement test, while factors B, D and P were the same as the AP test. The negative control, positive control and blank control were glucose, heparin and deletion serum without sample, respectively.

### 2.9. Statistical Analysis

All data were represented as mean ± standard deviation of three separate experiments. Statistical difference analysis and Pearson correlation analysis were carried out on SPSS 17.0 (SPSS Inc, Chicago, IL, USA). The network analysis of structure-activity correlation was visualized by Cytoscape 3.9.1 (National Institute of General Medical Sciences, Bethesda, MD, USA).

## 3. Results and Discussion

### 3.1. Preparation of Polysaccharides

**Figure 1:**
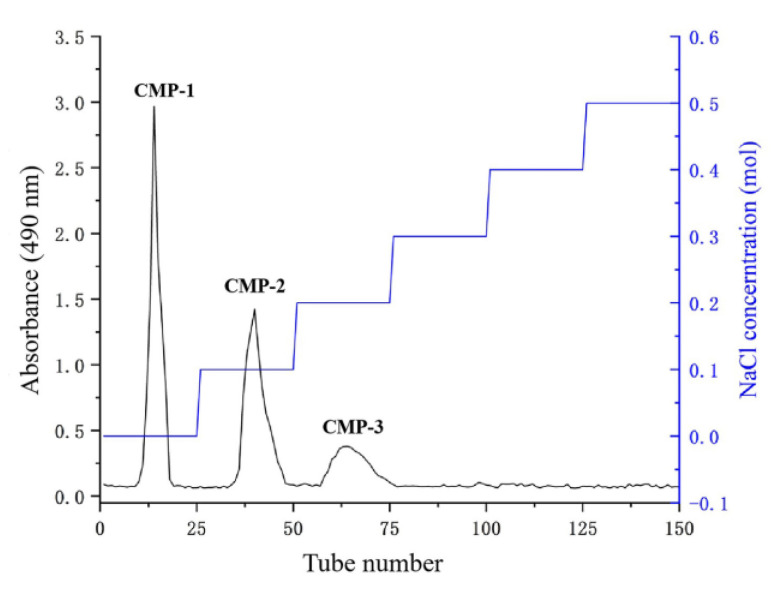
Elution profile of polysaccharides on DEAE-52 cellulose column.

The crude CMPs were prepared by hot water extraction followed by ethanol precipitation and freeze-drying, and then isolated using a DEAE-52 cellulose column (Figure 1). The three major polysaccharides peaks (CMP-1, CMP-2 and CMP-3) were obtained, which were eluted by the deionized water, 0.1 and 0.2 mol/L NaCl solution, respectively. The polysaccharide contents of CMP-1, CMP-2 and CMP-3 were 91.78 ± 2.11%, 92.95 ± 1.17% and 94.18 ± 2.18%, respectively. The yields of CMP-1, CMP-2 and CMP-3 were 24.31 ± 1.45%, 16.52 ± 1.04% and 10.02 ± 0.52%, respectively. The water soluble index of CMP-1, CMP-2 and CMP-3 was 98.67 ± 0.62%, 98.56 ± 0.83% and 97.85 ± 0.72%, respectively.

### 3.2. Analysis of Molecular Weights

**Figure 2:**
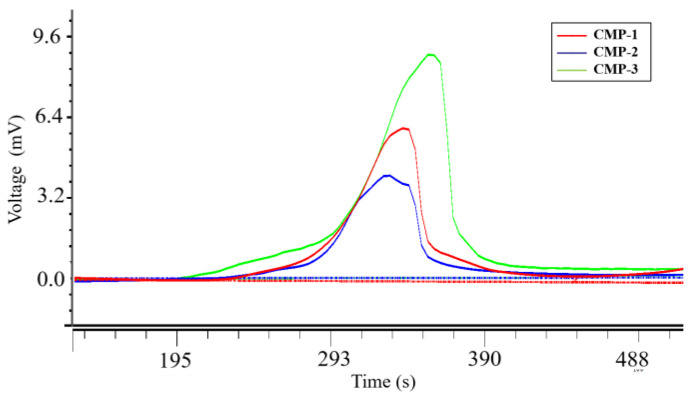
High performance gel permeation chromatography of CMP-1, CMP-2 and CMP-3.

The HPGPC chromatogram of three polysaccharides are presented in Figure 2. They are all homogeneous polysaccharides demonstrated by their chromatographic curves exhibiting a single peak. The results of GPC software fitting and calculation showed that the molecular weights of CMP-1, CMP-2 and CMP-3 were 2.19 × 10^6^ Da, 2.80 × 10^6^ Da and 1.74 × 10^6^ Da, respectively. Previous researchers have reported that the molecular weights of *C. militaris* polysaccharides were generally between ~10^3^ Da and ~10^5^ Da [30], and a kind of high molecular weight *C. militaris* polysaccharides (CMP-Ⅲ, 4.796×10^4^ kDa) was isolated by He et al. [31]. The molecular weights of CMP-1, CMP-2 and CMP-3 were observed to be significantly higher than those of the *C. militaris* polysaccharides that were previously reported, which may be considered as high molecular weight polysaccharides.

### 3.3. Analysis of Monosaccharide Compositions

**Figure 3:**
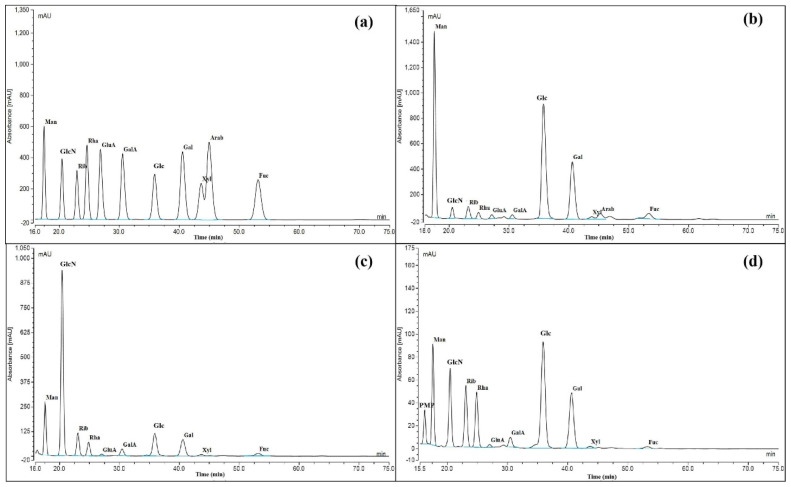
High performance liquid chromatography of standard monosaccharides (**a**), CMP-1 (**b**), CMP-2 (**c**) and CMP-3 (**d**). (Mannose: Man, glucosamine: GlcN, ribose: Rib, rhamnose: Rha, glucuronic acid: GlcA, galacturonic acid: GalA, glucose: Glc, galactose: Gal, xylose: Xyl, arabinose: Ara, fucose: Fuc).

The monosaccharide composition chromatograms of the three polysaccharides and their molar ratios are shown in Figure 3 and Table 1. Compared with the monosaccharide component of CMP-1, no trace of Ara was detected in CMP-2 and CMP-3. Previous studies have shown that *C. militaris* polysaccharides generally contained 3 to 7 kinds of monosaccharides, which were mainly composed of Man, Glc and Gal with different molar ratios [30]. Obviously, the CMP-1, CMP-2 and CMP-3 were composed of at least 10 kinds of different monosaccharides, which were more abundant than the previously reported polysaccharides from *C. militaris*. Compared with polysaccharides from other sources (including other fungi, algae and plants) [32,33], they were usually composed mainly of glucose and galactose in their monosaccharide composition. However, glucosamine in these three polysaccharides was rarely found in polysaccharides from plants.

**Figure 4:**
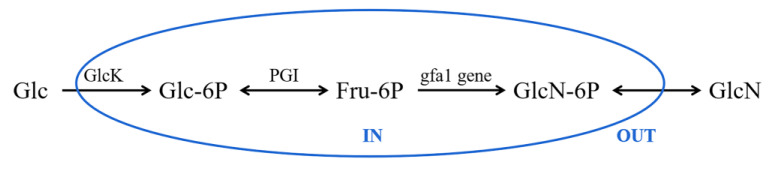
The putative biosynthetic pathway of glucosamine in *C. militaris* polysaccharides.

It is noteworthy that the three polysaccharides were also found to be composed of glucosamine, which were isolated from *C. militaris* for the first time. GlcN is an amino sugar, which was obtained by replacing the -OH group of the Glc molecule with an -NH_2_ group. Previous investigations demonstrated that glucosamine is widely distributed in fungi [34], and glucosamine-containing polysaccharides were also found in edible fungi, such as Helvella leucopus, Poria cocos and Lentinus edodes [35,36,37]. The biosynthetic pathway of glucosamine in *C. militaris* was speculated as follows (Figure 4): under the action of glucokinase (GlcK), extracellular glucose of *C. militaris* generates glucose-6-phosphate (Glc-6P), and further generates fructose-6-phosphate (Fru-6P) with the catalysis of phosphoisomerase (PGI) [38]. Glucosamine was synthesized by Fru-6P with glucosamine synthetase (gfa1 gene), and the glucosamine-containing polysaccharides were synthesized by dehydration condensation with other monosaccharides. Therefore, the three polysaccharides linked with glucosamine were novel *C. militaris* polysaccharides, and their speculated biosynthetic pathway was reasonable.

### 3.4. Analysis of FT-IR Spectra

**Figure 5:**
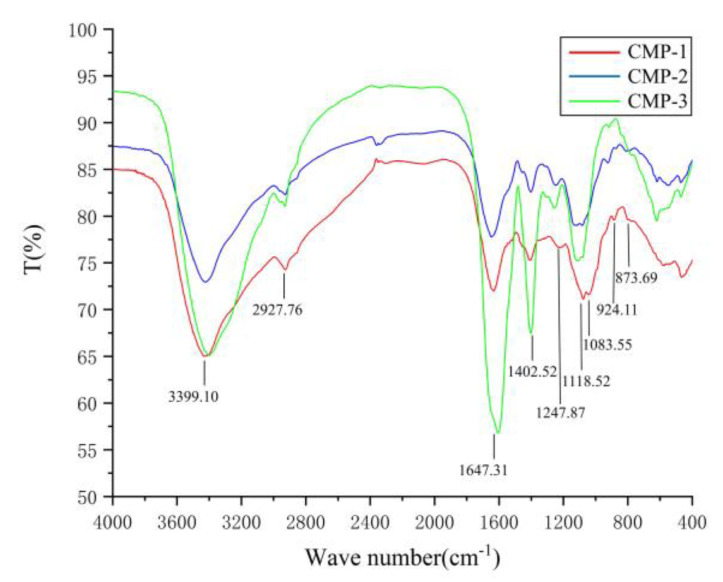
The FT-IR spectrum of CMP-1, CMP-2 and CMP-3.

The CMP-1, CMP-2 and CMP-3 possessed similar characteristic absorption peaks of polysaccharides (Figure 5). The absorption peaks in the range of 3180–3700 cm^−1^ might be associated with O-H stretching and N-H stretching vibrations, where the -OH and -NH_2_ groups were characteristic of the polysaccharides and its attached GlcN, respectively [39]. The weak absorption at 2927 cm^−1^ may be caused by the methyl group on Fuc or Rha [40]. The peak at 1650 cm^−1^ and 1402 cm^−1^ were related to associated water and bending vibrations of C-H in the sugar ring, respectively [41]. The peaks at 1000–1200 cm^−1^ are attributed to the existence of the pyranose ring [42]. The peaks at 924.11 cm^−1^ and 873.69 cm^−1^ showed the presence of *β*-and *α*-type glycosidic linkage in the three polysaccharides [43].

**Figure 6:**
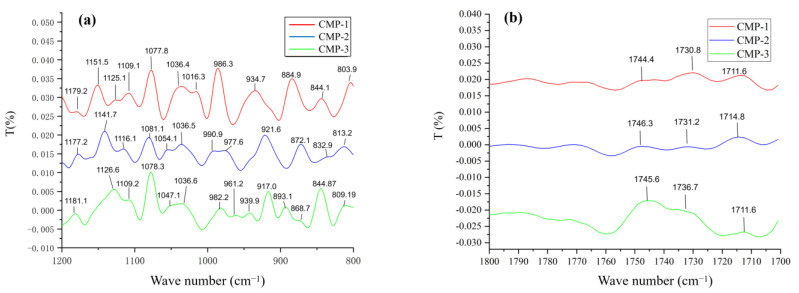
The second derivative FT-IR spectrum of three polysaccharides in the range of 800~1200 cm^−1^ (**a**) and 1700 ~ 1800 cm^−1^ (**b**).

Moreover, the additional frequency band information of the three polysaccharides was obtained by the second derivative of infrared spectrum, which proved that CMP-1, CMP-2 and CMP-3 were of significant difference in the fingerprint region (800 ~ 1300 cm^−1^) (Figure 6a). In detail, CMP-1 has three weak absorption peaks (1109 cm^−1^, 1125 cm^−1^, 1151 cm^−1^) in the range of 1110 ~ 1160 cm^−1^, which may be attributed to side chains on α-carb\zon. Compared with CMP-1, however, there was no obvious absorption peak at 1125 cm^−1^ in CMP-2 and 1150 cm^−1^ in CMP-3, under the condition of neglecting the weak wave number shift caused by the tolerance error. The same situation was evident within the range of 990~860 cm^−1^; CMP-3 had six absorption peaks, while CMP-1 had only three broad absorption bands and CMP-2 had only four absorption peaks. Therefore, the second derivative proved that significant differences existed among the three polysaccharides in the fingerprint region. In particular, the weak absorption peak of 1730^−1^ cm in the second derivative spectrum (Figure 6b) suggested that they contained a small amount of uronic acid [44], which was consistent with the results of the monosaccharide composition.

### 3.5. Congo Red Test

**Figure 7:**
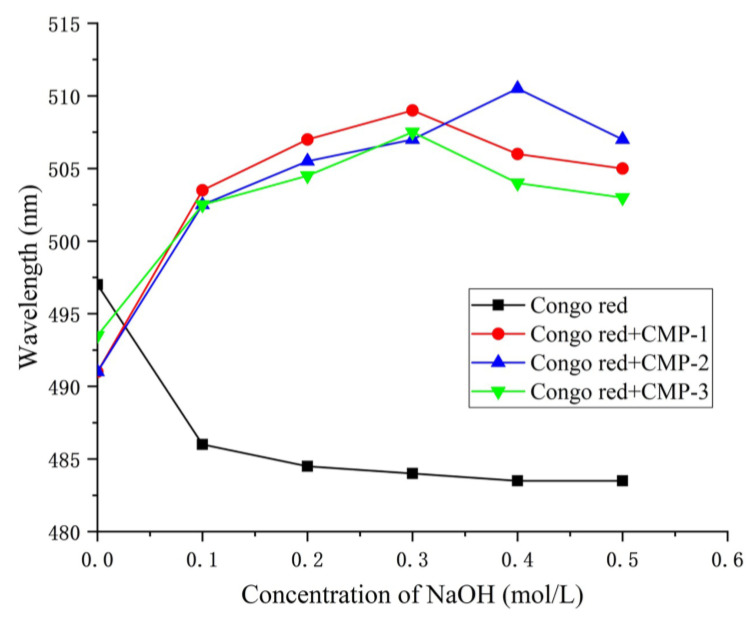
Change of maximum absorption wavelength of Congo red and three polysaccharide complexes.

Polysaccharides containing the triple helix conformation can form complexes and increased *λ_max_* with Congo red in weakly alkaline solutions, while in strongly alkaline solutions the helix is disrupted, leading to a decrease in the *λ_max_* of the complex [45]. As shown in Figure 7, the λ_max_ of Congo red + CMP-1, Congo red + CMP-2 and Congo red + CMP-3 increased with the increasing concentration of NaOH from 0 to 0.3 mol/L. Subsequently, the concentration of NaOH continued to increase, and the helix coil of the polysaccharide changed, indicating that the three polysaccharides all had the triple helix conformation.

### 3.6. Anti-Complementary Activity of CMP-1, CMP-2 and CMP-3

**Figure 8:**
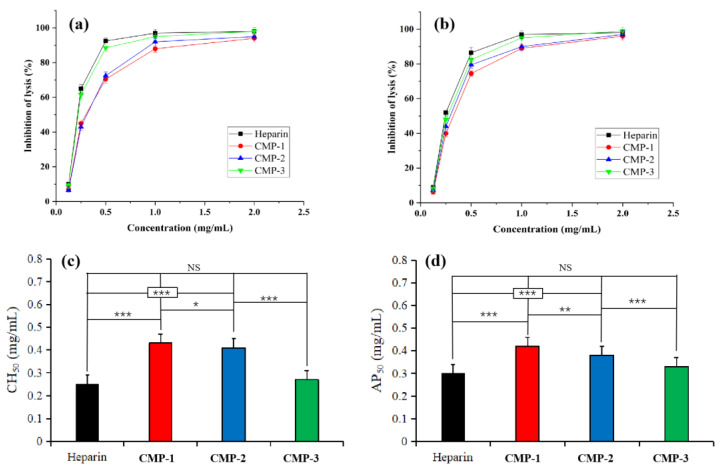
The anti-complementary activity of three polysaccharides with different dilution concentrations through CP (**a**) and AP (**b**). Comparison of CH_50_ (**c**) and AP_50_ (**d**). (NS: not significant, * *p* < 0.05, ** *p* < 0.01 and *** *p* < 0.001).

As shown in Figure 8a,b, CMP-1, CMP-2 and CMP-3 inhibited the hemolysis via classical pathway (CP) and alternative pathway (AP) in a dose-dependent manner within a concentration of 0.125 ~ 2.00 mg/mL. Furtherly, the half inhibitory concentration of the three polysaccharides on complement activation was calculated (Table 2), and the CH_50_ and AP_50_ differences between different samples were evaluated via one-way ANOVA analysis. As illustrated in Figure 8c,d, no significant difference (*p* > 0.05) was present between the two groups in CMP-3 (CH_50_ = 0.27 ± 0.04 mg/mL, AP_50_ = 0.33 ± 0.07 mg/mL) and heparin (CH_50_ = 0.25 ± 0.02 mg/mL, AP_50_ = 0.30 ± 0.03 mg/mL) on CP and AP, which indicated that CMP-3 had a similar anti-complementary effect with the positive drug. Although CMP-1 and CMP-2 can inhibit the activation of complement, the CH_50_ and AP_50_ values of CMP-1 (CH_50_ = 0.43 ± 0.07 mg/mL, AP_50_ = 0.42 ± 0.08 mg/mL) and CMP-2 (CH_50_ = 0.41 ± 0.08 mg/mL, AP_50_ = 0.38 ± 0.09 mg/mL) are significantly higher than those of heparin (*p* < 0.05), and the CH_50_ and AP_50_ values between CMP-1, CMP-2 and CMP-3 also showed significant differences (*p* < 0.05). The increasing order of complement inhibitory activity was as follows: CMP-1 < CMP-2 < CMP-3. These results indicated that the three polysaccharides have anti-complement activity via CP and AP, and CMP-3 had the strongest complement inhibition ability, which was worthy of further study.

**Figure 9:**
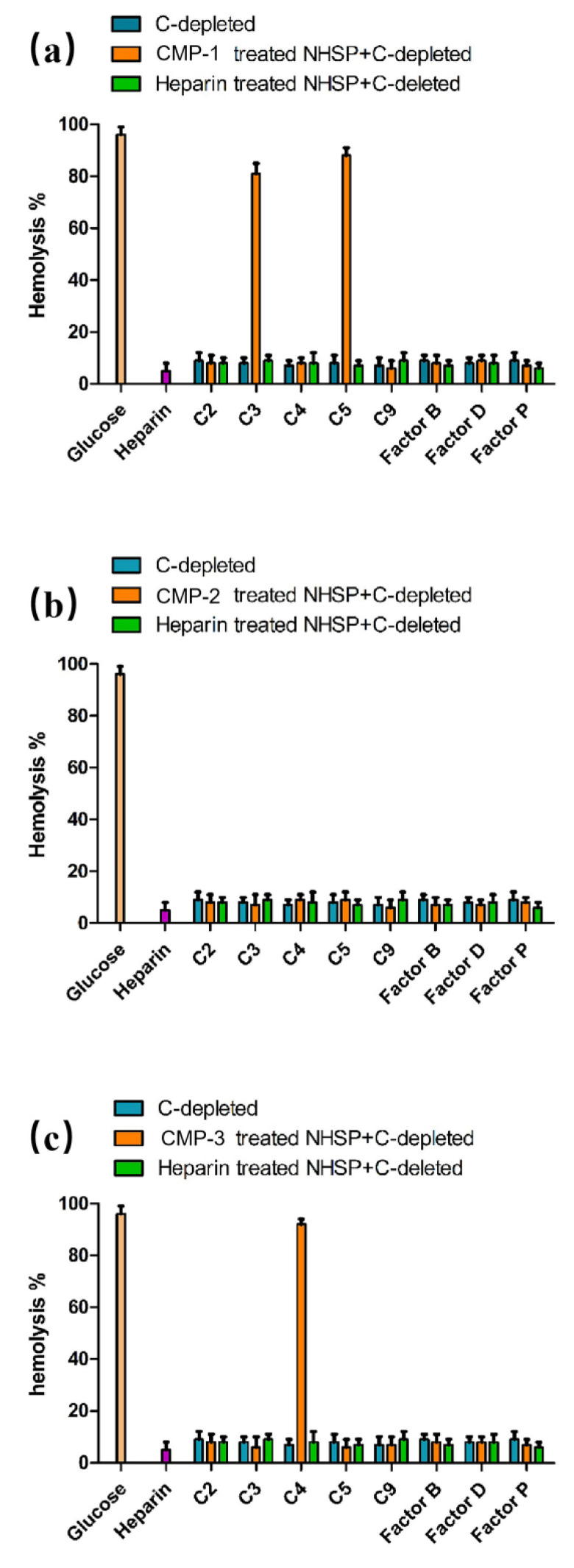
The targets of CMP-1 (**a**), CMP-2 (**b**) and CMP-3 (**c**) in the complement activation cascade.

The targets of polysaccharides action were determined based on the changes in hemolysis rates, reflected by the depletion of different complement components with CMP-1, CMP-2 and CMP-3. If the polysaccharides interact with the complement component being measured, it can exhibit hemolysis that cannot be recovered [46]. As shown in Figure 9, none of the complement depleted sera independently lysed red blood cells, and their hemolytic percentages were not more than 20%. After treatment with CMP-1, the complement-depleted sera of C2, C4, C9, Factor B, Factor D and Factor P did not restore hemolytic, while the C3- and C5-depleted sera significantly restored hemolytic activity. This proved that CMP-1 was inferred to block the activation cascade of the complement system by targeting C2, C4, C9, Factor B, Factor D and Factor P, but not with C3 and C5. Similarly, Figure 9b showed that CMP-2 can inhibit complement activation by interacting with C2, C3, C4, C5, C9, Factor B, Factor D and Factor P. Additionally, Figure 9c illustrates that CMP-3 can inhibit complement activation by interacting with C2, C3, C5, C9, Factor B, Factor D and Factor P, but not with C4.

### 3.7. Correlation analysis of structure and anti-complementary activity

**Figure 10:**
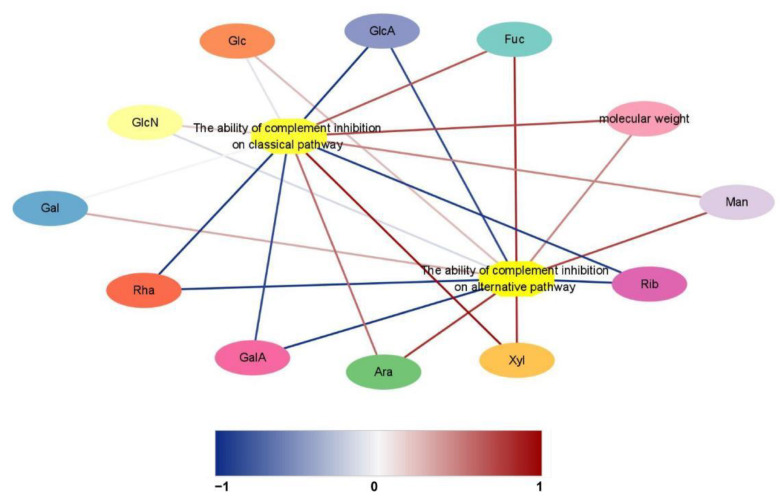
Network analysis of monosaccharide composition and molecular weight associated with the anti-complementary activity of CMPs. Red lines represent the positive correlation between structure and activity (*r* > 0.99). Blue lines represent the negative correlation between structure and activity (*r* > 0.99). The darker the line color, the higher is the significance of the correlation between structure and bioactivity.

The Pearson correlation is a more commonly used method for calculating correlation coefficients. It is one of the most fundamental approaches for revealing the direction and degree of linear correlation between two variables, and this correlation analysis technique has been used to explore the structure and bioactivity relationships of polysaccharides in recent years [47,48,49]. The correlation between the monosaccharide composition, molecular weight and anti-complementary activity of CMPs was analyzed, and the results and visualizations are shown in Table 3 and Figure 10. The structure activity correlation increased with the absolute value of Pearson’s correlation coefficient (*r*). In other words, the correlation coefficient values close to −1/1 suggested a stronger correlation between structural characterization and anti-complementary activity. Conversely, the correlation coefficient close to 0 was weak. The Rib and GalA composition of CMPs were significantly negatively correlated with the ability of the complement inhibition on the alternative pathway (*p* < 0.05). The correlation coefficients were −0.999 and −0.990, respectively. Meanwhile, a notable linear correlation was found between the GlcA composition and the ability of the complement inhibition on the classical pathway, and the correlation coefficient was −0.990 (*p* < 0.05). Although other monosaccharides showed different degrees of correlation with the anti-complementary activity of CMPs, the correlation was not significant (*p* > 0.05).

## 4. Conclusions

In the present study, the three polysaccharides (CMP-1, CMP-2 and CMP-3) were isolated from *C. militaris* for the first time. The results of monosaccharide composition, molecular weight, characteristic groups and stereo conformation showed that CMP-1, CMP-2 and CMP-3 were homogenous polysaccharides, composed of at least 10 monosaccharides with different molar ratios, and exhibited a triple helix structure with pyranoid polysaccharides. In addition, these three polysaccharides can interact with different complement components, and thus exhibited strong complement inhibitory activity. This laid the theoretical basis for the clinical application of *C. militaris* polysaccharides as novel anti-complementary drugs in the therapeutic process. The correlation analysis between the anti-complementary activity and polysaccharides showed that ribose, glucuronic acid and galacturonic acid played a major role in the complement inhibitory ability of *C. militaris* polysaccharides, which provided a new way to investigate the structure–activity relationship of *C. militaris* polysaccharides. However, the type and sequence of glycosidic linkage of CMP-1, CMP-2 and CMP-3 are unknown. Therefore, more analytical techniques, such as gas chromatography-mass spectrometry and nuclear magnetic resonance, can be used to investigate their structures in future research. The mechanism of biological activity in vivo needs to be further studied.

## Figures and Tables

**Table 1:** The molar ratios of monosaccharide composition of CMP-1, CMP-2 and CMP-3.

Samples	Man	GlcN	Rib	Rha	GlcA	GalA	Glc	Gal	Xyl	Ara	Fuc
CMP-1	39.35	4.03	3.98	2.56	1.62	1.52	70.52	26.90	1.00	3.23	4.23
CMP-2	13.62	86.70	7.80	6.22	1.47	2.99	17.80	9.26	1.00	ND	3.02
CMP-3	33.61	44.67	27.34	31.84	7.32	8.39	102.23	38.27	1.00	ND	5.79

Note: “ND” Not detected.

**Table 2:** The anti-complementary activity of CMP-1, CMP-2 and CMP-3.

Samples	CH_50_ (mg/mL)	AP_50_ (mg/mL)
CMP-1	0.43 ± 0.07	0.42 ± 0.08
CMP-2	0.41 ± 0.08	0.38 ± 0.09
CMP-3	0.27 ± 0.04	0.33 ± 0.07
Heparin	0.25 ± 0.02	0.30 ± 0.03

**Table 3:** Pearson correlation analysis of monosaccharide composition, molecular weight and anti-complementary activity of CMPs.

Value	Pearson	The Ability of Complement Inhibition on Classical Pathway-CH_50_	The Ability of Complement Inhibition on Alternative Pathway-AP_50_
Man	*r*	0.492	0.757
	*p*	0.336	0.226
GlcN	*r*	0.193	−0.149
	*p*	0.438	0.452
Rib	*r*	−0.954	−0.999 *
	*p*	0.097	0.013
Rha	*r*	−0.987	−0.983
	*p*	0.052	0.058
GlcA	*r*	−0.990 *	−0.886
	*p*	0.044	0.154
GalA	*r*	−0.885	−0.990 *
	*p*	0.154	0.044
Glc	*r*	−0.092	0.250
	*p*	0.471	0.420
Gal	*r*	−0.012	0.327
	*p*	0.492	0.394
Xyl	*r*	0.976	0.845
	*p*	0.070	0.175
Ara	*r*	0.596	0.832
	*p*	0.297	0.187
Fuc	*r*	0.680	0.888
	*p*	0.262	0.152
molecular weight	*r*	0.748	0.480
	*p*	0.231	0.341

Note: * *p* < 0.05; The absolute value of *r* = 0.8–1.0, extremely strong correlation; the absolute value of *r* = 0.6–0.8, strong correlation; the absolute value of *r* = 0.4–0.6, moderate correlation; the absolute value of *r* = 0.2–0.4, weak correlation; the absolute value of *r* = 0–0.2, extremely weak correlation or no correlation.

## Data Availability

The data presented in this study are available on request from the corresponding author.
